# MFGE8 promotes adult hippocampal neurogenesis in rats following experimental subarachnoid hemorrhage via modifying the integrin β3/Akt signaling pathway

**DOI:** 10.1038/s41420-024-02132-x

**Published:** 2024-08-11

**Authors:** Zhen-Yan Li, Xian Yang, Ji-Kai Wang, Xiao-Xin Yan, Fei Liu, Yu-Chun Zuo

**Affiliations:** 1 Department of Neurosurgery, Xiangya Hospital, Central South University, Changsha, 410008 China; 2 Department of Dermatology, The First Hospital of Hunan University of Chinese Medicine, Changsha, 410007 China; 3 Department of Neurosurgery, The Fifth Affiliated Hospital of Sun Yat-sen University, Zhuhai, 519000 China; 4 Department of Anatomy and Neurobiology, Xiangya School of Medicine, Central South University, Changsha, 410013 China

**Keywords:** Stroke, Neural stem cells

## Abstract

Subarachnoid hemorrhage (SAH) is one of the most severe type of cerebral strokes, which can cause multiple cellular changes in the brain leading to neuronal injury and neurological deficits. Specifically, SAH can impair adult neurogenesis in the hippocampal dentate gyrus, thus may affecting poststroke neurological and cognitive recovery. Here, we identified a non-canonical role of milk fat globule epidermal growth factor 8 (MFGE8) in rat brain after experimental SAH, involving a stimulation on adult hippocampal neurogenesis(AHN). Experimental SAH was induced in Sprague-Dawley rats via endovascular perforation, with the in vivo effect of MFGE8 evaluated via the application of recombinant human MFGE8 (rhMFGE8) along with pharmacological interventions, as determined by hemorrhagic grading, neurobehavioral test, and histological and biochemical analyses of neurogenesis related markers. Results: Levels of the endogenous hippocampal MFGE8 protein, integrin-β3 and protein kinase B (p-Akt) were elevated in the SAH relative to control groups, while that of hippocalcin (HPCA) and cyclin D1 showed the opposite change. Intraventricular rhMGFE8 infusion reversed the decrease in doublecortin (DCX) immature neurons in the DG after SAH, along with improved the short/long term neurobehavioral scores. rhMGFE8 treatment elevated the levels of phosphatidylinositol 3-kinase (PI3K), p-Akt, mammalian target of rapamycin (mTOR), CyclinD1, HPCA and DCX in hippocampal lysates, but not that of integrin β3 and Akt, at 24 hr after SAH. Treatment of integrin β3 siRNA, the PI3K selective inhibitor ly294002 or Akt selective inhibitor MK2206 abolished the effects of rhMGFE8 after SAH. In conclusion, MFGE8 is upregulated in the hippocampus in adult rats with reduced granule cell genesis. rhMFGE8 administration can rescue this impaired adult neurogenesis and improve neurobehavioral recovery. Mechanistically, the effect of MFGE8 on hippocampal adult neurogenesis is mediated by the activation of integrin β3/Akt pathway. These findings suggest that exogenous MFGE8 may be of potential therapeutic value in SAH management.

**Figure:**
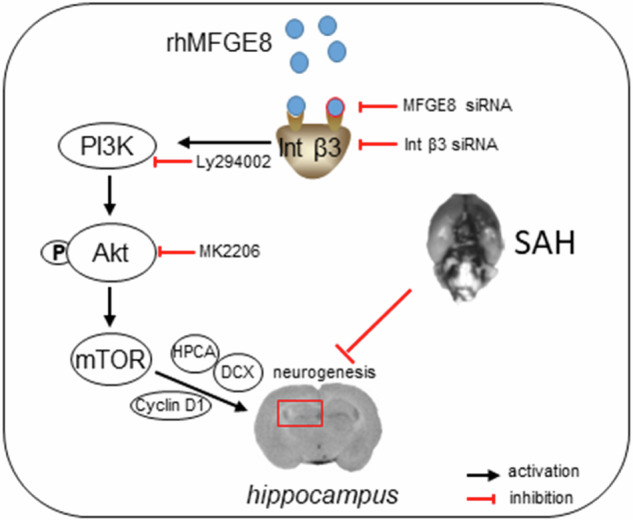
Graphical abstract and proposed pathway of rhMFGE8 administration attenuate hippocampal injury by improving neurogenesis in SAH models. SAH caused hippocampal injury and neurogenesis interruption. Administered exogenous MFGE8, recombinant human MFGE8(rhMFGE8), could ameliorate hippocampal injury and improve neurological functions after SAH. Mechanistically, MFGE8 bind to the receptor integrin β3, which activated the PI3K/Akt pathway to increase the mTOR expression, and further promote the expression of cyclin D1, HPCA and DCX. rhMFGE8 could attenuated hippocampal injury by improving neurogenesis after SAH, however, know down integrin β3 or pharmacological inhibited PI3K/Akt by ly294002 or MK2206 reversed the neuro-protective effect of rhMFGE8.

Graphical abstract and proposed pathway of rhMFGE8 administration attenuate hippocampal injury by improving neurogenesis in SAH models. SAH caused hippocampal injury and neurogenesis interruption. Administered exogenous MFGE8, recombinant human MFGE8(rhMFGE8), could ameliorate hippocampal injury and improve neurological functions after SAH. Mechanistically, MFGE8 bind to the receptor integrin β3, which activated the PI3K/Akt pathway to increase the mTOR expression, and further promote the expression of cyclin D1, HPCA and DCX. rhMFGE8 could attenuated hippocampal injury by improving neurogenesis after SAH, however, know down integrin β3 or pharmacological inhibited PI3K/Akt by ly294002 or MK2206 reversed the neuro-protective effect of rhMFGE8.

## Introduction

Subarachnoid hemorrhage (SAH) is a common type of hemorrhagic cerebral strokes [1], clinically associated with high mortality and disability [2] or poor prognostic outcome, causing a high socioeconomic burden worldwide [3–5]. As with all types of brain stroke, SAH can induce early brain injury (EBI) and delayed brain injury that contribute to poststroke neurological deficits, death or long-term disability. Notably, cerebral strokes can cause hippocampal neuronal injury, including impairment of adult neurogenesis in the dentate gyrus (DG), which may also relate to poor cognitive recovery among stroke survivors [6, 7].

Many animal studies show that adult hippocampal neurogenesis (AHN) plays an essential role in learning, memory and other cognitive functions [6, 8, 9]. Residual neural stem cells or progenitor cells at the subgranular zone (SGZ) of the DG proliferate, differentiate and mature, and eventually integrate into neural circuit to perform hippocampus-dependent neurological and cognitive functions [10]. A pivot role of AHN in maintenance or recovery of cognitive functions has been emphasized in many disease conditions such as cerebral stroke, traumatic brain injury and age-related neurodegenerative diseases [6, 7, 11, 12].

The milk fat globule-epidermal growth factor 8 (MFGE8) is a soluble glycoprotein characterized structurally by two N-terminal epidermal growth factor (EGF)-like domains. One of the EGF-like domain contains an integrin-binding motif, which binds to integrin β3 on the cell surface of phagocytes. As such, previous studies have focused a principal role of MFGE8 in the process of apoptosis. Recent study shows that MFGE8 is enriched in quiescent neural stem cells [13], and may play a role in mediating neural stem/progenitor cell proliferation and migration, including via angiogenesis [14].

The phosphatidylinositol 3-kinase (PI3K)/Akt (protein kinase B) and the mammalian target of rapamycin (mTOR) signaling pathways are involved in cell proliferation, growth and survival under physiological as well as pathological conditions. Evidence implicates that PI3K/Akt signaling pathway may act downstream to MFGE8 in the brain [15]. MFGE8 promotes C2C12 cell proliferation through the PI3K/Akt signaling pathway, while mTOR acts downstream to Akt, and modulate cell survival and apoptosis, cell cycle regulation and protein homeostasis. Notably, mTOR can modulate neurosphere growth and differentiation via an epigenetic mechanism [16, 17], pointing to an in vivo role of the PI3K/Akt/mTOR pathway in AHN. The differentiation of neural precursors into neurons is important for the neurological recovery in conditions such as brain injury and strokes. Hippocalcin (HPCA), a calcium-binding protein, appears to be involved in neuronal genesis or development. Specifically, it may participate in neuronal differentiation by inhibiting neural stem cells differentiating into astrocytic fate [18].

Currently, little is known whether, and if so, the underlying molecular signaling thereof, MFGE8 may regulate AHN affected by brain injuries such as following cerebral stroke. In the present study, we first confirmed the upregulation of endogenous MFGE8 in the hippocampus after experimental SAH. We further explored a role of this protein in AHN and the underlying signaling mechanism, through multiple approaches including intraventricular infusion of recombinant human MFGE8 (rhMFGE8), by in vivo silencing of MGFE8 expression, and by pharmacological intervention of the integrin β3/Akt /HPCA pathway.

## Results

### Overall assessment on animal mortality, SAH modeling and severity

**Fig. 1:**
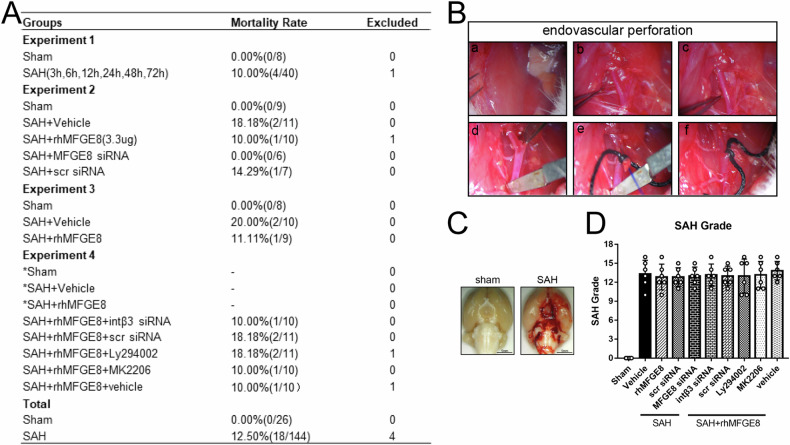
Animal mortality, endovascular perforation and general observation for SAH. **A** The animal usage and mortality rate in each group. **B** The procedure of SAH modeling by endovascular perforation method. **C** Representative brain sample images for sham and SAH. The blood clots mainly distributed around the Circle of Wills and brain stem after SAH. **D** SAH grade between groups in the experiment.

A total of 174 rats were used in the current study, including 4 rats that were excluded because of the SAH grade score <8. The mortality rate of the remaining 170 rats was listed in Fig. 1A, among which 26 rats received sham operation and 144 rats underwent SAH induction (Fig. 1B). The total mortality rate of SAH groups was 12.50% (18/144), which showed no significant difference among the SAH subgroups, while no animal died in the sham groups. The blood clot in the SAH animals was mainly distributed around the Circle of Wills and brain stem, whereas no blood was seen on the brain in sham groups (Fig. 1C). In addition, the means of SAH grades showed no statistically different among the SAH groups or SAH+rhMFGE8 groups (Fig. 1D).

### Expression of endogenous MFGE8 and HPCA in hippocampus after SAH

**Fig. 2:**
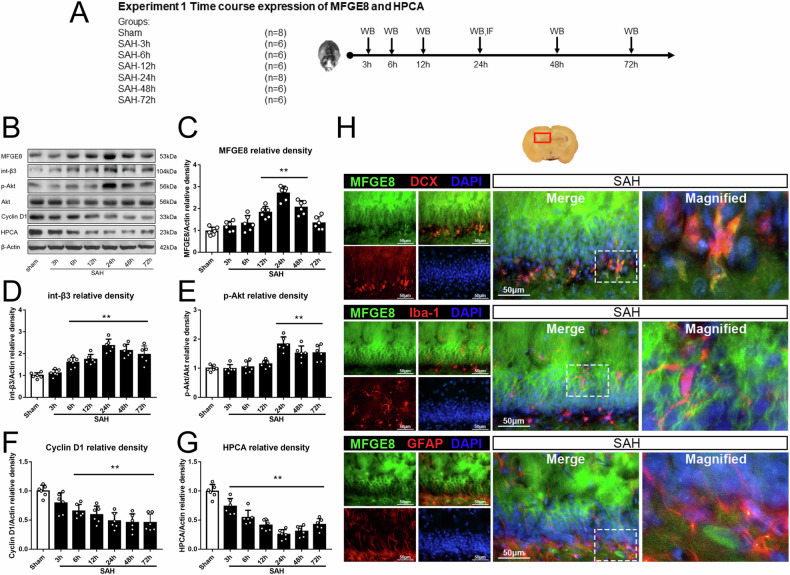
Temporal expression of endogenous MFGE8 and HPCA, and cellular localization of MFGE8 after SAH. **A** Experimental design, animal groups and time frame of the analyses in experiment 1. **B** Representative bands and (**C**–**G**). quantitative analyses of endogenous MFGE8 integrin β3, p-Akt, Akt, cyclin D1 and HPCA in left hippocampus after SAH. The MGFE8, integrin β3 and p-Akt are increased and peaked at 24 h after SAH, while cyclin D1 and HPCA show the opposite trend. **P < 0.01 vs sham group. Error bars were represented as mean ± SD. One-way ANOVA, Tukey’s test, *n* = 6 per group. **H** Double immunofluorescence staining of MFGE8 (green) with DCX (immature neuron marker, red), Iba-1(microglia marker, red) and GFAP (astrocytes marker, red) in hippocampus at 24 hr after SAH. Red box indicated the hippocampus in the brain slice. *n* = 2 per group. Scale bar, 50 μm. DCX doublecortin, Iba-1 ionized calcium binding adapter molecule-1, GFAP glial fibrillary acidic protein, DAPI diamidino phenylindole.

Western blot was performed to detect the expression of endogenous MFGE8 and HPCA in the hippocampus of animal groups with sham operation and SAH surviving 3, 6, 12, 24, 48 and 72 hr (Fig. 2A). The levels of MFGE8, integrin-β3, p-Akt started to increase at 12 hr after SAH and peaked at 24 hr. In contrast, the levels of cyclin D1 and HPCA showed an opposite trend, i.e., decreased after SAH induction to the lowest level at 24 hr post-surgery, then slowly recovered until 72 hr, although remained significant difference at this time point as compared to sham group (Fig. 2B–G). Double immunofluorescence was performed to study the cellular localization of MFGE8 in hippocampus after SAH. The results confirmed a localization of MFGE8 with the immature neuronal marker DCX, the astrocytic marker GFAP and the microglial marker Iba-1 in the hippocampal formation (Fig. 2H), indicting an expression of MFGE8 in multiple neuronal and glial cell phenotypes.

### rhMFGE8 promoted neurogenesis and short-term neurobehavioral performant after SAH

**Fig. 3:**
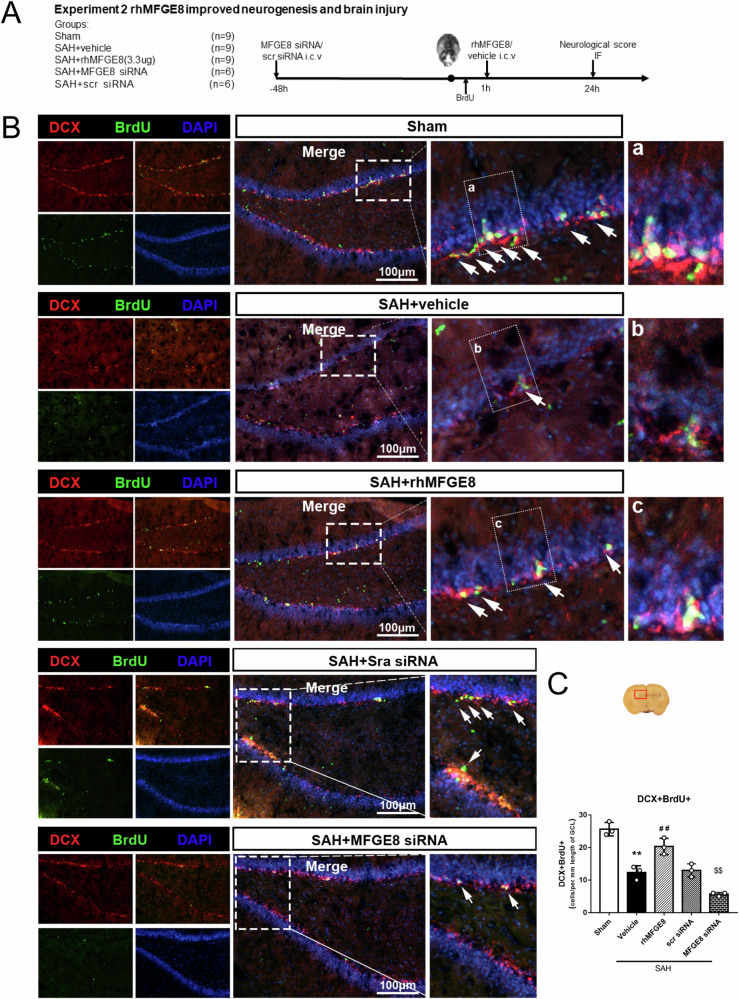
Effect of rhMFGE8 application or MGFE8 siRNA on hippocampal neurogenesis after SAH. **A** Experimental design, animal groups and time frame of the analyses in experiment 2. **B** Double immunofluorescence staining with DCX (red) and BrdU (green) in hippocampus between sham, SAH + vehicle and SAH + rhMFGE8 group as well as SAH + Scr siRNA, SAH + MFGE8 siRNA group. Arrows indicated the DCX positive and BrdU positive cells. **C** Quantitative analyses of DCX positive BrdU positive cells in three groups. The DCX positive BrdU positive cells are decreased in SAH+vehicle group compared to sham, which are reversed by rhMFGE8 treatment. The cells are also decreased in SAH + MFGE8 siRNA group compared to SAH + Scr siRNA group. The Red box indicated the hippocampus in the brain slice. **P < 0.01 vs sham group, ^##^*P* < 0.01 vs SAH + vehicle group, ^$$^*P* < 0.01 vs SAH + Scr siRNA group. Error bars were represented as mean ± SD. One-way ANOVA, Tukey’s test, *n* = 3 per group. Scale bar, 100 μm. BrdU bromodeoxyuridine.

**Fig. 4:**
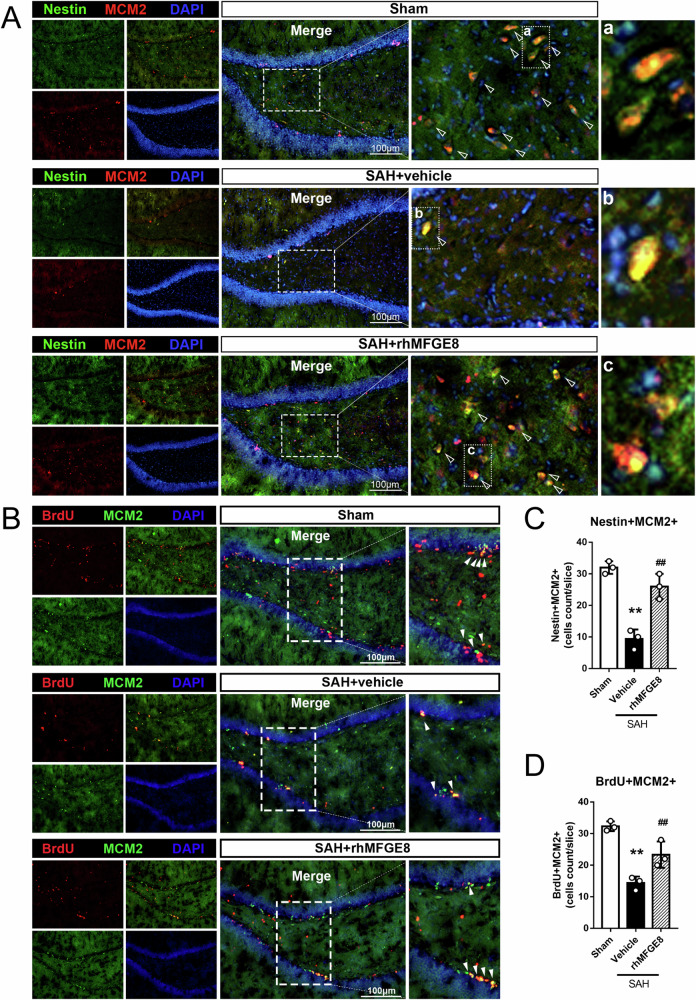
Effect of rhMFGE8 application on hippocampal neural stem/progenitor cells after SAH. **A** Double immunofluorescence staining with Nestin(green) and MCM2(red) in hippocampus between sham, SAH + vehicle and SAH + rhMFGE8 group. Triangles indicated the nestin positive and MCM2 positive cells. **B** Double immunofluorescence staining with BrdU(red) and MCM2(green) in hippocampus between sham, SAH + vehicle and SAH+rhMFGE8 group. Arrowheads indicated the MCM2 positive and BrdU positive cells. **C**, **D** Quantitative analyses of Nestin positive MCM2 positive cells and BrdU positive MCM2 positive cells in three groups. **P < 0.01 vs sham group, ^##^*P* < 0.01 vs SAH + vehicle group, Error bars were represented as mean ± SD. One-way ANOVA, Tukey’s test, *n* = 3 per group. Scale bar, 100 μm. MCM2, minichromosome maintenance complex component 2.

Short-term neurobehavioral deficits were evaluated by modified Garcia score and beam balance test at 24 hr after SAH between rhMFGE8 treated and control groups, with immunofluorescence used to assess the effect specifically on hippocampal adult neurogenesis (Fig. 3A). The numbers of DCX/BrdU double labeled cells in the dentate SGZ were significantly higher in the SAH+rhMFGE8 than SAH + vehicle groups (Fig. 3B). Consistent with the above results indicating a promotive effect of MFGE8 on hippocampal adult neurogenesis after SAH, the numbers of DCX/BrdU co-labeled cells at the SGZ were significantly lower in the SAH animals subjected an in vivo inhibition of MFGE8 expression by mRNA silencing (SAH + siRNA), relative to controls (SAH + scramble siRNA) (Fig. 3B, C). To further prove the results, another two markers nestin and MCM2 were performed to study neurogenesis. Nestin and MCM2 immunolabeled cells were significantly increased in the rhMFGE8 treated groups relative to control (Fig. 4A, C). Moreover, the numbers of BrdU/MCM2 double immunofluorescent cells were increased in the SAH groups treated with rhMFGE8 relative to those with vehicle (Fig. 4B, D).

### rhMFGE8 improved long-term recovery of neurological functions after SAH

**Fig. 5:**
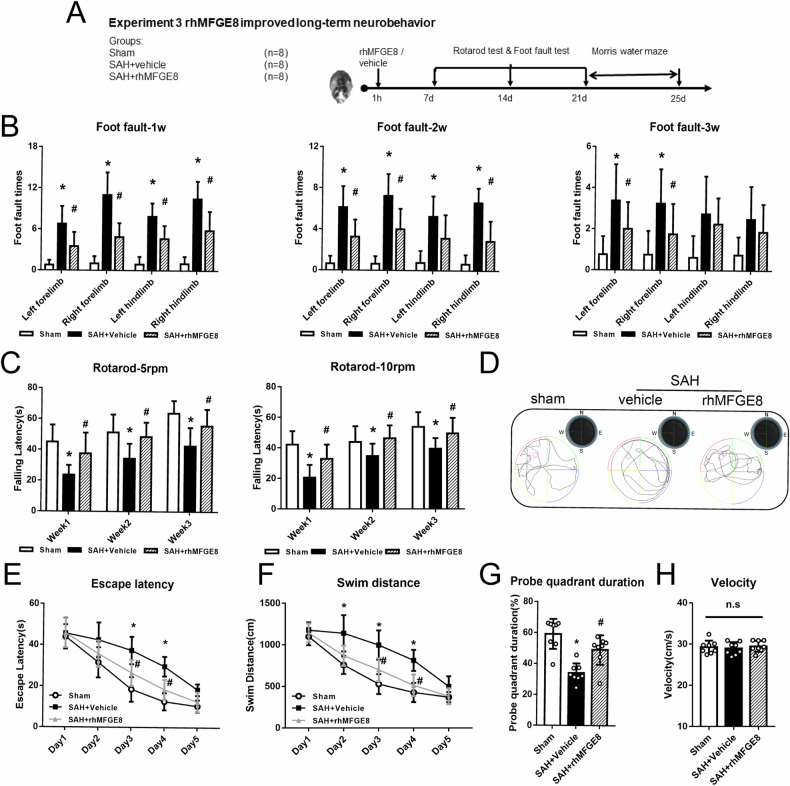
Long-term neurobehavioral effects of rhMFGE8 administration after SAH. **A** Experimental design, animal groups and time frame of the analyses in experiment 3. **B** Foot fault test at 1 to 3 week show that rhMFGE8 treated SAH animals have less foot fault times. **C** Rotarod test of 5 rpm and 10 rpm at 1–3 week show that rhMFGE8 treated SAH groups have longer falling latency. **D** Representative trace map in probe quadrant test showed rhMFGE8 treated SAH spent more time in target area. **E** Escape latency and (**F**). swim distance of water maze test from day 1 day 5 show that rhMFGE8 treated SAH animals have less escape latency and swimming distance to find the platform. **G** Probe quadrant duration in targeted area. **H** swim velocity of probe trial test. *P < 0.05, vs. sham group; ^#^*P* < 0.05, vs^.^ SAH + vehicle group. Data was represented as mean ± SD. Two-way ANOVA, *n* = 8 per group.

Foot fault and rotarod tests were performed to evaluate sensorimotor coordination and balance of the animals on days 7, 14 and 21, respectively after SAH (Fig. 5A). The times of four-limb fault in the rhMFGE8 treated SAH groups were significantly less than the group received vehicle at 7 days, even at 21 days (Fig. 5B). The SAH + vehicle group showed a markedly shorter falling latency in rotarod test at both 5 rpm and 10 rpm accelerating velocity when compared to sham operation group on days 7, 14 and 21 after SAH. In the rhMFGE8 treated groups, the falling latency was mitigated at both 5 rpm and 10 rpm velocities as compared to SAH + vehicle group (Fig. 5C). Similar beneficial effects of rhMFGE8 administration were seen in Morris water maze and probe quadrant tests reflecting spatial learning and memory capability. The trace map showed that the rats in SAH + vehicle group swam less distance than those in the sham or SAH + rhMFGE8 group (Fig. 5D). The escape latency and swim distance to find the target plate were markedly prolonged in the SAH+vehicle group compared with sham group. However, both the escape latency and swim distance were shortened on days 3–4 in SAH + rhMFGE8 group as compared to the vehicle treated group (Fig. 5E, F). In the probe quadrant test, after the target plate was removed from the water, the percentage of time spent in the target area was notably diminished in SAH + vehicle group compared to sham group. However, the duration in the SAH + rhMFGE8 group was significantly increased relative to the SAH + vehicle group (Fig. 5G). It should be noted that the swimming speed showed no significantly difference among the three groups (Fig. 5H).

### Interfering integrin β3/PI3K/Akt signaling disrupted the neuroprotection by rhMFGE8

**Fig. 6:**
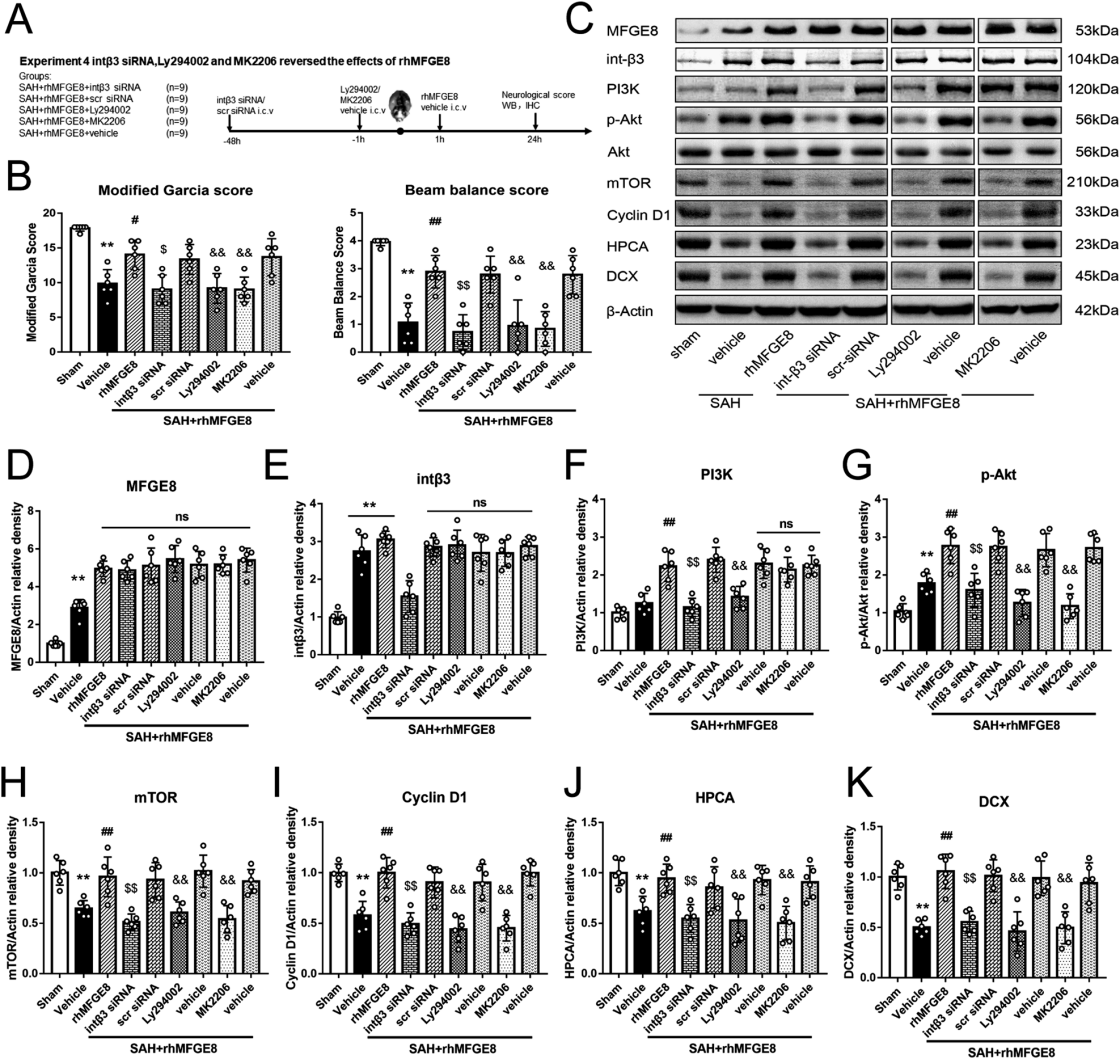
Knockdown integrin β3 or blocked PI3K/Akt signaling pathway abolished the rhMFGE8 treatment effects on neurological function. **A** Experimental design, animal groups and time frame of the analyses in experiment 4. **B** Modified Garcia score and beam balance score show that rhMFGE8 treatment improves the scores, with the effect reversed by int-β3 siRNA and PI3K/Akt inhibitor. **C** Representative western blot bands and (**D**–**K**). Quantitative analyses of MFGE8, int β3, PI3K, *p*-Akt, Akt, mTOR, cyclin D1, HPCA and DCX at 24 hr after SAH. **P < 0.01 vs sham group; ^##^*P* < 0.01 vs^.^ SAH+vehicle group; ^$$^ *P* < 0.01 vs. SAH + rhMFGE8+ scr siRNA; ^&&^ *P* < 0.01 vs. SAH+rhMFGE8 + vehicle^.^ Data were represented as mean ± SD. One-way ANOVA, Tukey’s test, *n* = 6 per group.

Using mRNA silencing and pharmacological intervening approaches, we further explored the involvement of the integrin β3/PI3K/Akt pathway in modulating the neuroprotective effect by MFGE8 after SAH. Neurobehavioral functions were examined in SAH animals subjected to intraventricular injection of integrin β3 siRNA at 48 hr and intraperitoneal administration of the specific PI3K inhibitor ly294002 or Akt inhibitor MK2206 at 1 hr before SAH induction, respectively, relative to controls (Fig. 6A). Both the Garcia score and beam balance scores were poorer in the SAH + rhMFGE8 + integrin β3 siRNA group relative to the SAH + rhMFGE8 + scramble siRNA group, and in the SAH + rhMFGE8 + ly294004 or SAH + rhMFGE8 + MK2206 group relative to the SAH + rhMFGE8 + vehicle group (Fig. 6B). The immunoblot data confirmed the effects of mRNA silencing and pharmacological inhibition on protein expression. Thus, comparing to vehicle control, rhMFGE8 treatment elevated the protein levels of integrin β3 and PI3K, *p*-Akt, mTOR and cyclin D1, HPCA, DCX in the hippocampal lysates. However, integrin β3 knockdown and PI3K or *p*-Akt pharmacological inhibition reversed the elevation of all the above proteins by rhMFGE8 administration in the SAH animals (Fig. 6C–K).

### Interfering integrin β3/PI3K/Akt signaling attenuated the enhanced neurogenesis by rhMFGE8

**Fig. 7:**
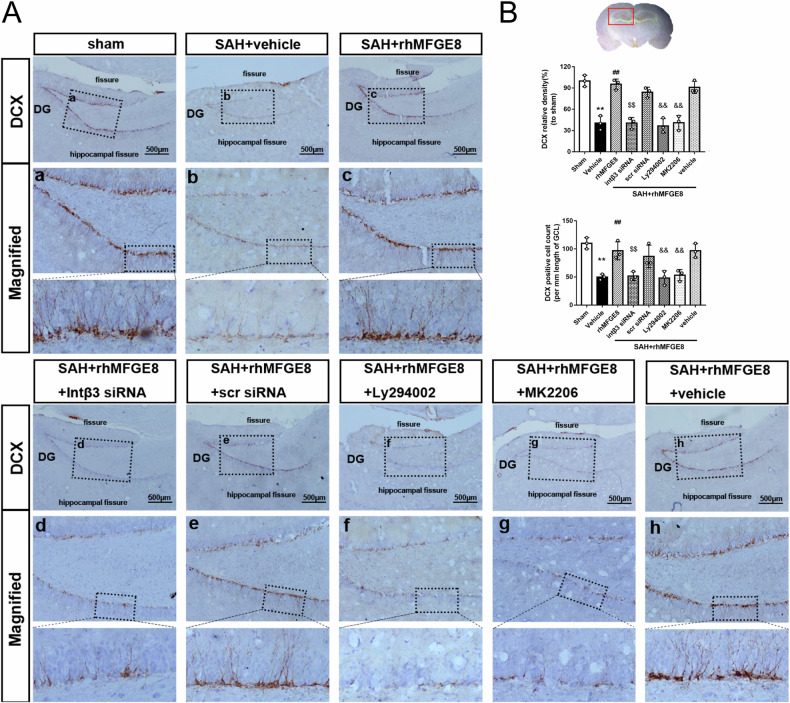
Knockdown integrin β3 or blocked PI3K/Akt signaling pathway abolished the effect of rhMFGE8 improving neurogenesis after SAH. **A** Immunohistochemical staining of DCX and (**B**). quantitative analyses of DCX relative density and DCX positive cell in the hippocampus. Red box indicated the hippocampus in the brain slice. **P < 0.01 vs sham group; ^##^*P* < 0.01 vs^.^ SAH + vehicle group; ^$$^ *P* < 0.01 vs. SAH + rhMFGE8+ scr siRNA; ^&&^ *P* < 0.01 vs. SAH + rhMFGE8 + vehicle^.^ Data were represented as mean ± SD. One-way ANOVA, Tukey’s test, *n* = 3 per group.

To confirm the attenuation of the rhMFGE8-induced hippocampal neurogenesis by mRNA silencing and pharmacological intervention of integrin β3/PI3K/Akt pathway, we further compared the amount of DCX-expressing immature neurons among the different treatment groups. In parallel with the alteration in the levels of DCX protein blotted in hippocampal lysates (as described above), the numerical density of DCX immunolabeled neurons in the dentate SGZ was greatly decreased in SAH + vehicle group, which was rescued in the SAH + rhMFGE8 group. However, the trend of recovery of DCX immunolabeled immature neurons by rhMFGE8 treatment was blunted by mRNA silencing of the receptor integrin β3 as well as by the inhibition of ly294002 or MK2206 on PI3K/Akt activation (Fig. 7A–B).

### Administered rhMFGE8 attenuated hippocampal injury after SAH

**Fig. 8:**
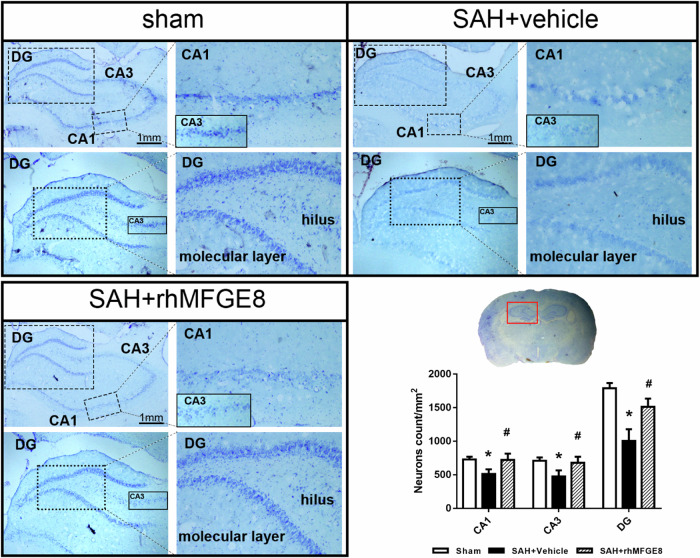
Administration of rhMFGE8 attenuated hippocampal injury after SAH. Nissl staining of hippocampus and quantitative analyses of Nissl staining in CA1, CA3 and dentate gyrus (DG). Red box indicated the hippocampus in the brain slice. *P < 0.05, vs. sham group; #*P* < 0.05, vs. SAH + vehicle group. Data was represented as mean ± SD. *n* = 3 per group.

Nissl staining of the brain slice were performed to confirm the neuroprotective effect of rhMFGE8 after SAH. The density of neurons in both cornu ammonis area 1 (CA1), CA3 and dentate gyrus (DG) were dramatically decreased in SAH + vehicle group compared to sham, which were reversed in SAH + rhMFGE8 group (Fig. 8). The result revealed that rhMFGE8 attenuated the hippocampal injury after SAH.

## Discussion

In the current study, we explored a protective effect of MFGE8 in vivo and the underlying molecular signaling thereof by improving AHN following experimental SAH in rats. The major findings could be summarized as the following. (1) Experimental SAH led to both short-term and long-term neurological and cognitive deficits in rats. (2) Levels of endogenous MFGE8 protein in the hippocampus was increased after SAH and peaked at 24 hr. The elevated MFGE8 expression was localized to immature neurons, as well as astrocytes and microglia. Levels of HPCA protein in hippocampus was decreased after SAH and reached to lowest at 24 hr. (3) Administration of exogenous rhMFGE8 improved short- and long-term neurological deficits, and mitigated the reduction of AHN after SAH. (4) rhMGFE8 treatment elevated the levels of PI3K, p-Akt, mTOR, Cyclin D1, HPCA and DCX in the hippocampus, although did not alter that of the integrin β3 and Akt protein. (5) Knockdown of integrin β3 by mRNA silencing or pharmacological inhibition of PI3K by Ly294002 or Akt by MK2206 attenuated the neurobehavioral protection and the impact on integrin β3/PI3K/Akt signaling by rhMGFE8. Taken together, our results indicate that MGFE8 is involved in the process of restoration of AHN after SAH, which may be important for neurological and cognitive recovery. This effect can be modulated by the integrin β3/PI3K/Akt signaling pathway. The findings also suggest that pharmacological administration of rhMFGE8 can accelerate recovery of neurobehavioral functions and neurogenesis in rats subjected to an experimental hemorrhagic insult.

Cerebral stroke including SAH can induce multiple traumatic effects resulting in early and delayed brain injuries. One of these harmful effects involves the inhibition of adult neurogenesis in the hippocampal formation, an important mechanism to support neuroplasticity underlying learning and memory as well as other cognitive and affective behaviors [13]. Previously we report that in the rat model of SAH, AHN is impaired, which is correlated to the occurrence of neurological deficit [19]. The inflammatory microenvironment induced by experimental SAH appears to play a role in the attenuation of neurogenesis in the dentate gyrus [20]. Therefore, promoting neurogenesis could be a putative strategy in the management of SAH.

MFGE8 has been known to regulate phagocytosis and promote engulfment of dying cells [21]. However, emerging evidence points to a protective cellular effect of this protein in response to traumatic insults such as ischemia-reperfusion injury (cutaneous, intestinal, renal, myocardial and brain) [22–24] and inflammation (sepsis, airway allergic, neurodegeneration) [25–27]. It also can promote tissue regeneration, including cutaneous angiogenesis and wound healing. Of note, MFGE8 appears to play a neuroprotective role in conditions such as ischemic and hemorrhagic cerebral stroke [28–30], and traumatic brain injury [15, 31].

In the present study we first verified the upregulation of endogenous MFGE8 in the hippocampus following the induction of SAH. Double immunofluorescence revealed the expression of MFGE8 in neural precursors and immature neurons, in agreement with previous transcriptome data that MFGE8 is enriched in pluripotent and neural stem cells in the adult rodent brain. In consideration of its potential clinical application, the human version of MFGE8 (rhMFGE8), which is structurally homogenous and functionally equivalent to murine MFGE8, was used to further explore a specific role of this protein in AHN after experimental stroke. We found that rhMFGE8 administration can increase DCX immature neurons as well as BrdU birth-dated neural stem cells expressing nestin and MCM2 in the hippocampus. These observation supports a notion that rhMFGE8 can promote the self-renewal of the neural precursor pool as well as the differentiation of newborn cells towards neuronal phenotype.

The PI3K/Akt pathway is crucial in cell survival and proliferation, while MFGE8 may serve as an upstream regulator of this signaling system by binding to the receptor integrins. For instance, MFGE8 reduces apoptosis via integrin β3/FAK/PI3K/Akt signaling pathway in rat model of traumatic brain injury [15]. MFGE8 can modulate M1/M2 microglial polarization and A1/A2 astrocytic alteration via NF-kB and PI3K/Akt pathways [27, 32]. Further, MFGE8 promotes cell proliferation through the PI3K/Akt pathway in C2C12 cells and hepatocytes [33, 34]. mTOR is a serine/threonine kinase protein that regulates quiescence/activation of neural stem cells [17]. Hippocalcin (HPCA) is a calcium-binding protein that can promote neuronal differentiation by inhibition of astrocytic differentiation [18, 35]. Therefore, we decided to determine explore whether MFGE8 may regulate AHN through the integrin β3/PI3K/ Akt/HPCA pathway. These findings obtained from experiments with in vivo rhMFGE8 administration, mRNA and pharmacological interfering suggest that MFGE8 appear to promote AHN by serving as an upstream regulator of the integrin β3/Akt signaling system (Fig. 8).

There are a number of limitations or issues remained to be further addressed in the present study. First, MFGE8 may also help maintenance of the dentate neural niche via angiogenesis [14], which is not specifically examined in current experiment. Second, astrocytes are known to modulate adult dentate neurogenesis via secreted factors [36–38]. Our previous study revealed that activated astrocytes and dentate neurogenesis showed the opposite change pattern after SAH. Therefore, it remains to clarify whether MFGE8 may affect dentate neurogenesis indirectly through astrocytic responses. Third, rhMFGE8 may also mitigate early brain injury by other effects, such as anti-inflammation and anti-apoptosis. Thus, the beneficial effect of rhMFGE8 on the recovery of neurobehavior could be better considered a sum of multiple mechanisms, which warrants further clarification. Finally, the role of MFGE8 could be further investigated using genetic approaches such as knock-out experiments.

In summary, the present study shows that MFGE8 expression is endogenously activated in the hippocampus in rats following experimental SAH. Administration of rhMFGE8 can promote hippocampal adult neurogenesis and improve the recovery of short- and long-term neurological deficit in animals suffered from SAH. Mechanistically, the beneficial role of MFGE in adult neurogenesis may relate to integrin β3/Akt signaling. These findings also point to a utility of rhMFGE8 in the treatment of SAH in clinical settings.

## Materials and methods

### Animals and surgery

Adult male Sprague–Dawley rats (Fig. 1A, *n* = 174; weighing 280–300 g) were obtained from the Laboratory Animal Center of Xiangya School of Medicine and housed in an animal vivarium with 12/12 hr light/dark illumination, constant temperature (25 °C) and humidity, and allowed free access to food and water. All the experiments and procedures were approved by the Ethics committee of Animal Care and Use at Central South University (No.CSU-2022–0301). The study was performed in accordance with the Declaration of Helsinki.

SAH modeling was performed using endovascular perforation as previously described [39, 40]. The main procedures were illustrated in Fig. 1B. Briefly, the rats were anesthetized and placed on a warm pad with a constant 37°C temperature during surgery. A midline incision on the neck was cut, followed by the exposure of the left common, internal and external carotid artery. A 3 cm-long prolene suture (4–0) with sharpened end was inserted into the external carotid artery. The suture was advanced into internal carotid artery to the bifurcation of the anterior and middle cerebral arteries, where a slight resistance was felt. At this point, a further advance (3 mm) was made to induce a vascular perforation before the suture withdrawn immediately. The rats in sham groups underwent the same surgical procedures except for the arterial perforation. The surgery was ended by closing the incision, and the animals were returned to their home cages after complete anesthesia recovery.

### Experimental design

Animals were randomly assigned into four separate experiments detailed below. The experimental design and time line of behavioral and brain examinations were integrated into corresponding figures. The information about experimental groups was blinded to the researchers who carried out the behavioral tests, western blot, immunofluorescence and data analysis.

Experiment 1: This was aimed to determine the time course of protein expression in the hippocampus. A total of 42 rats were divided randomly and assigned into 7 groups with *n* = 6/group: sham, SAH 3, 6, 12, 24, 48 and 72 hr (*n* = 6/group). The temporal trend of endogenous MFGE8, integrin-β3, protein kinase B (p-Akt), cyclin D1 and HPCA expression was detected by western blot. Four additional rats assigned as SAH and sham group (*n* = 2/group) surviving 24 hr were used for histological studies with double immunofluorescence.

Experiment 2: This was designed to assess the short-term neuroprotective effect of recombinant human MFGE8(rhMFGE8) on hippocampal neurogenesis and neurological recovery after SAH. A total of 27 rats were randomized into 3 groups (*n* = 9/group): sham, SAH + vehicle, SAH+ rhMFGE8 (3.3 ug/kg). rhMFGE8 or vehicle was administered at 1 h after SAH induction by intracerebroventricular (i.c.v) injection, using a dosage based on our previous studies [41]. To label newborn cells, BrdU (50 mg/kg, Abcam, USA) was injected intraperitoneally (i.p) half an hour following SAH induction. Modified Garcia score and beam balance test were measured at 24 hr after SAH. In addition, a totally of 12 rats were divided into SAH + MGFE8 siRNA (*n* = 6) and SAH+scramble siRNA (*n* = 6) groups, to evaluate the effect of endogenous MFGE8 on AHN with SAH.

Experiment 3: This experiment was set to carry out long-term neurobehavioral tests, including foot fault test, rotarod test and Morri’s water maze test, in animals with and without rhMFGE8 administration. A total of 24 rats were randomly divided into 3 groups (*n* = 8/group): sham, SAH + vehicle, SAH + rhMFGE8 (3.3 ug/kg). The foot fault and rotarod tests were performed on days 7, 14 and 21, and water maze on days 21–25, after induction of SAH.

Experiment 4: This experiment was to determine the involvement of integrin β3/PI3K/Akt/mTOR pathway in rhMFGE8 mediated effect on neurogenesis after SAH. The integrin β3 siRNA (int β3 siRNA) was used to silence the receptor, while Ly294002 (Selleck, USA) or Akt selective inhibitor MK2206 (Selleck, USA) was to pharmacologically block the activity of PI3K or Akt. These agents were administrated via i.c.v. infusion at 48rs and 1 hr before SAH, respectively. A total of 45 rats were randomly divided into 5 groups (*n* = 9/group): SAH + rhMFGE8+ int β3 siRNA, SAH + rhMFGE8+ scr siRNA, SAH + rhMFGE8 + ly294002, SAH + rhMFGE8 + MK2206, SAH+rhMFGE8+vehicle. Data from the sham, SAH + vehicle, SAH + rhMFGE8 groups from Experiment 2 were also included for collective analysis (to avoid unnecessary animal use). Neurobehavioral performance, western blot and immunofluorescence/immunohistochemistry were assessed at 24 hr after SAH.

### Reagent preparation and application

rhMFGE8 (R&D systems, USA) was diluted in sterile saline to a concentration of 0.33 ug/μl. An equal dose of rhMFGE8 (3.3 ug) was administered to all animals 1 h after SAH by i.c.v injection [29, 41], while animals in the control group received an equal volume (10 μl) of saline. MFGE8 siRNA (10 μl, concentration of 5000 IFU/μl, SantaCruz, USA), integrin β3 siRNA (10 μl, concentration of 5000 IFU/μl, SantaCruz, USA) or scrambled siRNA (10 μl, concentration 5000 IFU/μl, Santa Cruz, USA) were administered by i.c.v at 48 hr before SAH [41]. BrdU (50 mg/kg, abcam, USA) was diluted in sterile saline and administered by intraperitoneal (i.p) injection half an hour after SAH [19]. Ly294002 (50 mmol/L, Selleck, USA) and MK2206 (42 mmol/L, Selleck, USA) was diluted in 10% DMSO and administered by i.c.v injection 1 h before SAH [42, 43]. The dosage of the above reagents were previously optimized by our group and others.

### Intracerebroventricular injection

Intracerebroventricular drug administration was carried out as descripted previously [44, 45]. Briefly, the anesthetized rat was placed in a stereotaxic apparatus (RWD, China) on a prone position. A craniotomy was drilled on the right side of skull at 1.5 mm posterior and 1.0 mm lateral relative to bregma. A 10 μl syringe (Microliter701; Hamilton Company, Switzerland) was inserted through the cranial hole to a depth of 3.3 mm below cranial surface. The injection was slowly proceeded during a period of 10 min, with the needle maintained for an additional 3 min, followed by a slow withdrawal over a period of 2 min.

### SAH severity assessment

The severity of SAH was assessed using a previously established grading method [46]. After removal from the skull, the brain was photographed on the ventral view. Using the documented digital images, the basal cistern on each brain was divided into six parts and extent of bleeding thereof was scored respectively according to amount of subarachnoid blood clots: 0, no subarachnoid blood; 1, minimal subarachnoid clots; 2, moderate subarachnoid clots with recognizable arteries; 3, blood clots covering arteries unrecognizable. The SAH grade score for each brain was the sum by addition of the scores of all six parts. Rats with mild SAH (SAH grade score <8) at 24 hr were excluded from experimental analysis.

### Short-term neurological assessment

Short-term neurological function was assessed with modified Garcia and beam balance tests at 24 hr after SAH, as previously described [47]. The modified Garcia test was scored based on six individually motor functions, including spontaneous activity, spontaneous movement of four limbs, body proprioception, whisker proprioception, forepaw outstretching and climbing. The overall Garcia score for a given animal was calculated by summing up all sub-scores of six tests, ranging from 3 to 18 [48]. Beam balance test was performed to observe the ability of rats walking on a narrow beam during a period of 60 s, which was scored based on the following criteria: 0, no walking and falling; (1) no walking, but remains on the beam; (2) walking but falling; (3) walking <20 cm; and 4, walking beyond 20 cm.

### Long-term neurological function assessment

Long-term neurological performance was assessed based on foot fault test, the rotarod test and Morri’s water maze test, as previously reported [49]. Briefly, foot fault test was performed by placing the rats on a horizontal grid floor. A foot fault was recorded if the rat placed an inaccurate limb and fell it through the grid. The result was recorded as foot fault times of every limb during 2 min. Rotarod test was performed to assess the balance and coordination abilities to stay on a rotating cylinder. Rats were placed on the rotarod at the starting speed of 5 revolutions per minute (rpm) and 10 rpm respectively, both were accelerated by 2 revolutions per 5 s. The duration of which the rats remained staying on the rotarod cylinder was recorded. For Morris water maze test, the rats were trained to find a visible platform slightly higher than water level in 60 s after random placing them in four different start locations. A probe trail was performed on the last day after 5 days continuous training, with the platform removed. For formal tests, swim trace map, escape latency and swim distance to find the platform as well as probe quadrant duration were video-recorded by a computerized tracking system (Smart v3.0, Panlab Harvard apparatus, USA).

### Western blot

The hippocampi were dissected out after brain removal, with samples extracted by the RIPA buffer (Beyotime, Shanghai, China), followed by centrifuged with 14,000 g at 4 °C for 30 min. The supernatant was collected and preserved at −80 °C until use. Protein concentration of the supernatant was determined using the bicinchoninic acid kit (Beyotime, Shanghai, China). Equal amounts of protein samples were loaded and separated in 6%–12% sodium dodecyl sulfate–polyacrylamide (SDS-PAGE) gel, and transferred to polyvinylidene fluoride membranes (PVDF 0.45μm, Millipore, MA, USA). The membranes were blocked with non-fat milk for 2 hr at room temperature, and then incubated overnight at 4 °C with the following primary antibodies: mouse anti-MFGE8 (1:1,000, sc-271574; Santa Cruz, USA), rabbit anti-integrin β3 (1:1,000, ab119992; Abcam, USA), mouse anti-PI3K (1:1000, 67121-Ig; Proteintech, China), mouse anti-Akt (1:500, sc-377556; Santa Cruz, USA), mouse anti-p-Akt (1:500, sc-5298; Santa Cruz, USA), mouse anti-mTOR (1:500,sc-517464; Santa Cruz, USA), mouse anti-cyclin D1 (1:1,000, sc-8396; Santa Cruz, USA), rabbit anti-HPCA (1:1,000, ab24560; Abcam, USA), rabbit anti-DCX (1:1,000, ab18723; Abcam, USA) and mouse anti-β-Actin (1:2,000, sc47778; Santa Cruz, USA). After incubations with the appropriate horseradish peroxidase (HRP) conjugated secondary antibodies (1:3000, Santa Cruz, USA) at room temperature for 2 hr, the target protein bands in the membrane were visualized using an ECL kit (Amersham Biosciences, Pittsburgh, PA). The relative densities of immunoblotting bands were analyzed using the Image J software (Image J 1.4, NIH, USA).

### Immunohistochemistry and immunofluorescence

Rats were transcardially rinsed briefly with cold PBS and perfused with 4% paraformaldehyde under deep anesthesia (100 mg/kg, sodium pentobarbital, i.p). The brains were removed, post-fixed in the perfusion fixative at 4 °C for 24 hr, and cryoprotected through 15% to 30% sucrose. The forebrain was cut into 20-μm-thick coronal sections in a cryostat (CM3050S; Leica Microsystems, Buffalo Grove, USA), which were collected into 12 sets in culture plates. For immunohistochemistry, a set of sections were taken, first treated in 3% H_2_O_2_ in PBS for 30 min, and then incubated in 5% normal horse serum at room temperature for 1 hr. Sections were further reacted with rabbit anti-DCX (1:1000, ab18723, Abcam, USA) at 4 °C overnight. On the next day, the sections were incubated with biotinylated horse anti-rabbit IgG at 1:400 in PBS for 1 hr at room temperature, and further with avidin-biotin complex (ABC) (1:400; Vector Laboratories Inc., Burlingame, CA, USA). Immunoreactive product was visualized using 0.003% H2O2 and 0.05% 3, 3-diaminobenzidine (DAB). After dehydration and mounting, the sections were examined and imaged on an Olympus BX51 microscope.

For double immunofluorescence, the sections were treated with 50% formamide/2 × SSC at 65 °C for 1 hr, and 2 N HCl for 30 min at 37 °C to denature DNA, then rinsed 3 times in PBS, and further incubated in 5% normal donkey serum at m temperature for 1 hr. The sections were subsequently incubated overnight at 4 °C with rat anti-BrdU (1:500, ab6326; Abcam, USA) together with one of the following primary antibodies: mouse anti-MFGE8 (1:200, sc-271574; Santa Cruz, USA), rabbit anti-DCX (1:1,000, ab18723; Abcam, USA), rabbit anti-Iba-1 (1:1000, ab178847; Abcam, USA), rabbit anti-GFAP (1:1000, ab7260; Abcam, USA), rabbit anti-MCM2(1:500, 10513-AP; Proteintech, China), and mouse anti-Nestin (1:500, 66259–1-Ig; Proteintech, China). The immunoreactive signals were revealed by Alexa Fluor® 488 and Alexa Fluor® 594 conjugated donkey antibodies against rat, rabbit or mouse IgGs (1:200, Jackson ImmunoResearch, USA). The sections were counterstained with DAPI (1:2000, Beyotime, China) before mounted on glass slides with an anti-fading medium.

### Nissl staining

Brain sections were stained in 0.1% cresyl violet for 2 min. After rinsing with water, sections were dehydrated in increasing concentrations of ethanol and cleared in xylenes and then mounted with and coverslippered with the permount media. Sections were observed and imaged on an Olympus BX51.

### Quantification and statistical analysis

The sample size was based on our previous studies involving experimental SAH and verified using sample size calculator (SigmaPlot). The number of DCX + BrdU, Nestin + MCM2, BrdU + MCM2 positive cells in the hippocampus sections were counted in three consecutive coronal sections (spaced 200 um apart) per animal by moving along the subgranular zone (SGZ) of the dentate gyrus (DG). Results were recorded as the percent colocalized cells. To obtain the numerical densities of DCX+ neurons, the labeled somata were counted along the SGZ in both the dorsal and ventral blade of the DG, while the length of the GCL was measured using Image J (NIH, USA), with number of cells per unit (mm) length of GCL calculated for each brain sample [50, 51]. For western blot data, the optic densities of the specific protein bands were measured using the Image J software.

For statistical analyses, data from individual animal groups were expressed as mean and standard deviation (mean ± SD), and plotted using Graph Pad Prism 7 (Graph Pad Software, San Diego, CA). Statistical analyses were performed using SPSS 16.0 software (SPSS Inc., Chicago, Illinois, USA), with P < 0.05 considered as being significant different. Specifically, two-way ANOVA was applied to analyze long-term neurobehavioral results. Shapiro-Wilk test was used to test the normality, with variables were log transformed when necessary. Student *t* test was used to compare the continuous variables with normal distribution between two groups. One-way analysis of variance (ANOVA) followed by Tukey’s post hoc test was used to compare for multiple groups with continuous independent variables expressed by means ± SD.

### Supplementary information

Supplemental Materials

## Data Availability

All data generated or analyzed during this study are included in this published article.

## References

[1] Claassen J, Park S. Spontaneous subarachnoid haemorrhage. Lancet. 2022;400:846–62. 35985353 10.1016/S0140-6736(22)00938-2 PMC9987649

[2] van Gijn J, Kerr RS, Rinkel GJ. Subarachnoid haemorrhage. Lancet. 2007;369:306–18. 17258671 10.1016/S0140-6736(07)60153-6

[3] Lapchak PA, Zhang JH. The high cost of stroke and stroke cytoprotection research. Trans Stroke Res. 2017;8:307–17. 10.1007/s12975-016-0518-y PMC6452866 28039575

[4] Tu WJ, Zhao Z, Yin P, Cao L, Zeng J, Chen H, et al. Estimated burden of stroke in China in 2020. JAMA Netw Open. 2023;6:e231455. 36862407 10.1001/jamanetworkopen.2023.1455 PMC9982699

[5] Ma Q, Li R, Wang L, Yin P, Wang Y, Yan C, et al. Temporal trend and attributable risk factors of stroke burden in China, 1990-2019: an analysis for the global burden of disease study 2019. Lancet Public Health. 2021;6:e897–906. 34838196 10.1016/S2468-2667(21)00228-0 PMC9047702

[6] Ceanga M, Dahab M, Witte OW, Keiner S. Adult neurogenesis and stroke: a tale of two neurogenic niches. Front Neurosci. 2021;15:700297. 34447293 10.3389/fnins.2021.700297 PMC8382802

[7] Cuartero MI, Garcia-Culebras A, Torres-Lopez C, Medina V, Fraga E, Vazquez-Reyes S, et al. Post-stroke neurogenesis: friend or foe? Front Cell Dev Biol. 2021;9:657846. 33834025 10.3389/fcell.2021.657846 PMC8021779

[8] Hayashi Y, Jinnou H, Sawamoto K, Hitoshi S. Adult neurogenesis and its role in brain injury and psychiatric diseases. J Neurochem. 2018;147:584–94. 30028510 10.1111/jnc.14557

[9] Varadarajan SG, Hunyara JL, Hamilton NR, Kolodkin AL, Huberman AD. Central nervous system regeneration. Cell. 2022;185:77–94. 34995518 10.1016/j.cell.2021.10.029 PMC10896592

[10] Aragon MJ, Topper L, Tyler CR, Sanchez B, Zychowski K, Young T, et al. Serum-borne bioactivity caused by pulmonary multiwalled carbon nanotubes induces neuroinflammation via blood-brain barrier impairment. Proc Natl Acad Sci USA 2017;114:E1968–76. 28223486 10.1073/pnas.1616070114 PMC5347541

[11] Marzano LAS, de Castro FLM, Machado CA, de Barros J, Macedo ECT, Simoes ESAC, et al. Potential role of adult hippocampal neurogenesis in traumatic brain injury. Curr Med Chem. 2021;29:3392–3419. 10.2174/0929867328666210923143713 34561977

[12] Berger T, Lee H, Young AH, Aarsland D, Thuret S. Adult hippocampal neurogenesis in major depressive disorder and Alzheimer’s disease. Trends Mol Med. 2020;26:803–18. 32418723 10.1016/j.molmed.2020.03.010

[13] Zhou Y, Bond AM, Shade JE, Zhu Y, Davis CO, Wang X, et al. Autocrine Mfge8 signaling prevents developmental exhaustion of the adult neural stem cell pool. Cell Stem Cell. 2018;23:444–52.e444. 30174295 10.1016/j.stem.2018.08.005 PMC6128767

[14] Cheyuo C, Aziz M, Wang P. Neurogenesis in neurodegenerative diseases: role of MFG-E8. Front Neurosci. 2019;13:569. 31213977 10.3389/fnins.2019.00569 PMC6558065

[15] Gao YY, Zhang ZH, Zhuang Z, Lu Y, Wu LY, Ye ZN, et al. Recombinant milk fat globule-EGF factor-8 reduces apoptosis via integrin beta3/FAK/PI3K/AKT signaling pathway in rats after traumatic brain injury. Cell Death Dis. 2018;9:845. 30154436 10.1038/s41419-018-0939-5 PMC6113274

[16] Wang T, Medynets M, Johnson KR, Doucet-O’Hare TT, DiSanza B, Li W, et al. Regulation of stem cell function and neuronal differentiation by HERV-K via mTOR pathway. Proc Natl Acad Sci USA 2020;117:17842–53. 32669437 10.1073/pnas.2002427117 PMC7395438

[17] Nieto-Gonzalez JL, Gomez-Sanchez L, Mavillard F, Linares-Clemente P, Rivero MC, Valenzuela-Villatoro M, et al. Loss of postnatal quiescence of neural stem cells through mTOR activation upon genetic removal of cysteine string protein-alpha. Proc Natl Acad Sci USA 2019;116:8000–9. 30926666 10.1073/pnas.1817183116 PMC6475374

[18] Park SY, Yoon SN, Kang MJ, Lee Y, Jung SJ, Han JS. Hippocalcin promotes neuronal differentiation and inhibits astrocytic differentiation in neural stem cells. Stem Cell Rep. 2017;8:95–111. 10.1016/j.stemcr.2016.11.009 PMC5233403 28017654

[19] Zuo Y, Wang J, Enkhjargal B, Doycheva D, Yan X, Zhang JH, et al. Neurogenesis changes and the fate of progenitor cells after subarachnoid hemorrhage in rats. Exp Neurol. 2019;311:274–84. 30359565 10.1016/j.expneurol.2018.10.011

[20] Zuo Y, Wang J, Liao F, Yan X, Li J, Huang L, et al. Inhibition of heat shock protein 90 by 17-AAG reduces inflammation via P2X7 receptor/NLRP3 inflammasome pathway and increases neurogenesis after subarachnoid hemorrhage in mice. Front Mol Neurosci. 2018;11:401. 30459553 10.3389/fnmol.2018.00401 PMC6232389

[21] Lemke G. How macrophages deal with death. Nat Rev Immunol. 2019;19:539–49. 31019284 10.1038/s41577-019-0167-y PMC6733267

[22] Uchiyama A, Yamada K, Perera B, Ogino S, Yokoyama Y, Takeuchi Y, et al. Protective effect of MFG-E8 after cutaneous ischemia-reperfusion injury. J Invest Dermatol. 2015;135:1157–65. 25493650 10.1038/jid.2014.515

[23] Howangyin KY, Zlatanova I, Pinto C, Ngkelo A, Cochain C, Rouanet M, et al. Myeloid-epithelial-reproductive receptor tyrosine kinase and milk fat globule epidermal growth factor 8 coordinately improve remodeling after myocardial infarction via local delivery of vascular endothelial growth factor. Circulation. 2016;133:826–39. 26819373 10.1161/CIRCULATIONAHA.115.020857 PMC4767109

[24] Brissette MJ, Laplante P, Qi S, Latour M, Cailhier JF. Milk fat globule epidermal growth factor-8 limits tissue damage through inflammasome modulation during renal injury. J Leukoc Biol. 2016;100:1135–46. 27260955 10.1189/jlb.3A0515-213RR

[25] Hansen LW, Yang WL, Bolognese AC, Jacob A, Chen T, Prince JM, et al. Treatment with milk fat globule epidermal growth factor-factor 8 (MFG-E8) reduces inflammation and lung injury in neonatal sepsis. Surgery. 2017;162:349–57. 28343695 10.1016/j.surg.2017.02.006 PMC5513803

[26] Khalifeh-Soltani A, Gupta D, Ha A, Podolsky MJ, Datta R, Atabai K. The Mfge8-alpha8beta1-PTEN pathway regulates airway smooth muscle contraction in allergic inflammation. FASEB J. 2018;15:fj201800109R. 10.1096/fj.201800109R 29763381

[27] Shi X, Cai X, Di W, Li J, Xu X, Zhang A, et al. MFG-E8 selectively inhibited abeta-induced microglial M1 polarization via NF-kappaB and PI3K-Akt pathways. Mol Neurobiol. 2017;54:7777–88. 27844286 10.1007/s12035-016-0255-y

[28] Cheyuo C, Jacob A, Wu R, Zhou M, Qi L, Dong W, et al. Recombinant human MFG-E8 attenuates cerebral ischemic injury: its role in anti-inflammation and anti-apoptosis. Neuropharmacology. 2012;62:890–900. 21964436 10.1016/j.neuropharm.2011.09.018 PMC3262883

[29] Liu F, Hu Q, Li B, Manaenko A, Chen Y, Tang J, et al. Recombinant milk fat globule-EGF factor-8 reduces oxidative stress via integrin beta3/nuclear factor erythroid 2-related factor 2/heme oxygenase pathway in subarachnoid hemorrhage rats. Stroke. 2014;45:3691–7. 25342030 10.1161/STROKEAHA.114.006635 PMC4245320

[30] Gao Y, Tao T, Wu D, Zhuang Z, Lu Y, Wu L, et al. MFG-E8 attenuates inflammation in subarachnoid hemorrhage by driving microglial M2 polarization. Exp Neurol. 2020;336:113532. 10.1016/j.expneurol.2020.113532 33245889

[31] Xiao Y, Li G, Chen Y, Zuo Y, Rashid K, He T, et al. Milk fat globule-epidermal growth factor-8 pretreatment attenuates apoptosis and inflammation via the integrin-beta3 pathway after surgical brain injury in rats. Front Neurol. 2018;9:96. 29535679 10.3389/fneur.2018.00096 PMC5834760

[32] Xu X, Zhang A, Zhu Y, He W, Di W, Fang Y, et al. MFG-E8 reverses microglial-induced neurotoxic astrocyte (A1) via NF-kappaB and PI3K-Akt pathways. J Cell Physiol. 2018;234:904–14. 30076715 10.1002/jcp.26918

[33] Li H, Xu W, Ma Y, Zhou S, Xiao R. Milk fat globule membrane protein promotes C2C12 cell proliferation through the PI3K/Akt signaling pathway. Int J Biol Macromol. 2018;114:1305–14. 29634969 10.1016/j.ijbiomac.2018.04.026

[34] Li H, Zhang T, Wang K, Lu M, Guo Y, Zhang Y, et al. MFGE8 protects against CCl4 -induced liver injury by reducing apoptosis and promoting proliferation of hepatocytes. J Cell Physiol. 2019;234:16463–74. 10.1002/jcp.28314 30767216

[35] Kang MJ, Park SY, Han JS. MicroRNA-24-3p regulates neuronal differentiation by controlling hippocalcin expression. Cell Mol Life Sci. 2019;76:4569–80. 31486848 10.1007/s00018-019-03290-3 PMC6841749

[36] Lie DC, Colamarino SA, Song HJ, Desire L, Mira H, Consiglio A, et al. Wnt signalling regulates adult hippocampal neurogenesis. Nature. 2005;437:1370–5. 16251967 10.1038/nature04108

[37] Cope EC, Gould E. Adult neurogenesis, glia, and the extracellular matrix. Cell Stem Cell. 2019;24:690–705. 31051133 10.1016/j.stem.2019.03.023 PMC7961263

[38] Asrican B, Wooten J, Li YD, Quintanilla L, Zhang F, Wander C, et al. Neuropeptides modulate local astrocytes to regulate adult hippocampal neural stem cells. Neuron. 2020;108:349–66.e346. 32877641 10.1016/j.neuron.2020.07.039 PMC7606593

[39] Siler DA, Gonzalez JA, Wang RK, Cetas JS, Alkayed NJ. Intracisternal administration of tissue plasminogen activator improves cerebrospinal fluid flow and cortical perfusion after subarachnoid hemorrhage in mice. Trans Stroke Res. 2014;5:227–37. 10.1007/s12975-014-0329-y PMC4012892 24526376

[40] Muroi C, Fujioka M, Mishima K, Irie K, Fujimura Y, Nakano T, et al. Effect of ADAMTS-13 on cerebrovascular microthrombosis and neuronal injury after experimental subarachnoid hemorrhage. J Thromb Haemost. 2014;12:505–14. 24476338 10.1111/jth.12511

[41] Wang J, Wang Y, Zuo Y, Duan J, Pan A, Li JM, et al. MFGE8 mitigates brain injury in a rat model of SAH by maintaining vascular endothelial integrity via TIGbeta5/PI3K/CXCL12 signaling. Exp Brain Res. 2021;239:2193–205. 33991211 10.1007/s00221-021-06111-x

[42] Peng J, Pang J, Huang L, Enkhjargal B, Zhang T, Mo J, et al. LRP1 activation attenuates white matter injury by modulating microglial polarization through Shc1/PI3K/Akt pathway after subarachnoid hemorrhage in rats. Redox Biol. 2019;21:101121. 30703614 10.1016/j.redox.2019.101121 PMC6351270

[43] Xie Z, Huang L, Enkhjargal B, Reis C, Wan W, Tang J, et al. Intranasal administration of recombinant Netrin-1 attenuates neuronal apoptosis by activating DCC/APPL-1/AKT signaling pathway after subarachnoid hemorrhage in rats. Neuropharmacology. 2017;119:123–33. 28347836 10.1016/j.neuropharm.2017.03.025 PMC5490977

[44] Iniaghe LO, Krafft PR, Klebe DW, Omogbai EKI, Zhang JH, Tang J. Dimethyl fumarate confers neuroprotection by casein kinase 2 phosphorylation of Nrf2 in murine intracerebral hemorrhage. Neurobiol Dis. 2015;82:349–58. 26176793 10.1016/j.nbd.2015.07.001 PMC4640980

[45] Chen S, Zhao L, Sherchan P, Ding Y, Yu J, Nowrangi D, et al. Activation of melanocortin receptor 4 with RO27-3225 attenuates neuroinflammation through AMPK/JNK/p38 MAPK pathway after intracerebral hemorrhage in mice. J Neuroinflam. 2018;15:106. 10.1186/s12974-018-1140-6 PMC5896146 29642894

[46] Altay O, Suzuki H, Hasegawa Y, Ostrowski RP, Tang J, Zhang JH. Isoflurane on brain inflammation. Neurobiol Dis. 2014;62:365–71. 24084689 10.1016/j.nbd.2013.09.016 PMC3919516

[47] Sugawara T, Ayer R, Jadhav V, Zhang JH. A new grading system evaluating bleeding scale in filament perforation subarachnoid hemorrhage rat model. J Neurosci Methods. 2008;167:327–34. 17870179 10.1016/j.jneumeth.2007.08.004 PMC2259391

[48] Liu L, Fujimoto M, Nakano F, Nishikawa H, Okada T, Kawakita F, et al. Deficiency of tenascin-C alleviates neuronal apoptosis and neuroinflammation after experimental subarachnoid hemorrhage in mice. Mol Neurobiol. 2018;55:8346–54. 10.1007/s12035-018-1006-z 29546590

[49] Xie Z, Enkhjargal B, Wu L, Zhou K, Sun C, Hu X, et al. Exendin-4 attenuates neuronal death via GLP-1R/PI3K/Akt pathway in early brain injury after subarachnoid hemorrhage in rats. Neuropharmacology. 2018;128:142–51. 28986282 10.1016/j.neuropharm.2017.09.040 PMC5714662

[50] Xiong K, Cai Y, Zhang XM, Huang JF, Liu ZY, Fu GM, et al. Layer I as a putative neurogenic niche in young adult guinea pig cerebrum. Mol Cell Neurosci. 2010;45:180–91. 20599617 10.1016/j.mcn.2010.06.009 PMC2923265

[51] Wan L, Huang RJ, Luo ZH, Gong JE, Pan A, Manavis J, et al. Reproduction-asociated hormones and adult hippocampal neurogenesis. Neural Plast. 2021;2021:3651735. 34539776 10.1155/2021/3651735 PMC8448607

