# Supplementary material for: MFGE8 promotes adult hippocampal neurogenesis in rats following experimental subarachnoid hemorrhage via modifying the integrin β3/Akt signaling pathway
Source: Cell Death Discov. 2024 Aug 11;10:359. doi: 10.1038/s41420-024-02132-x (PMC11317487; doi:10.1038/s41420-024-02132-x)

supplementary material

The original western blot bands listed below:

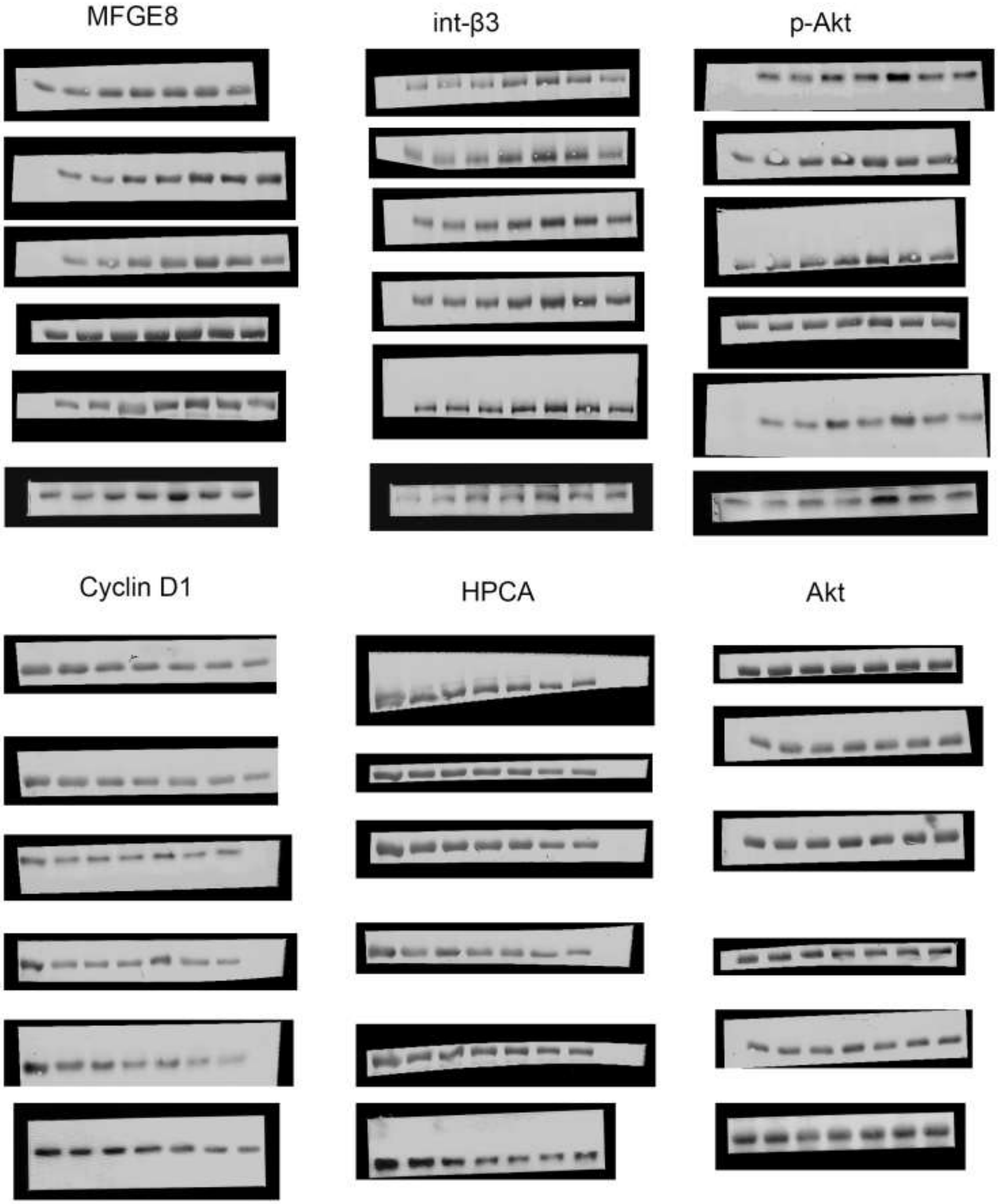

Actin

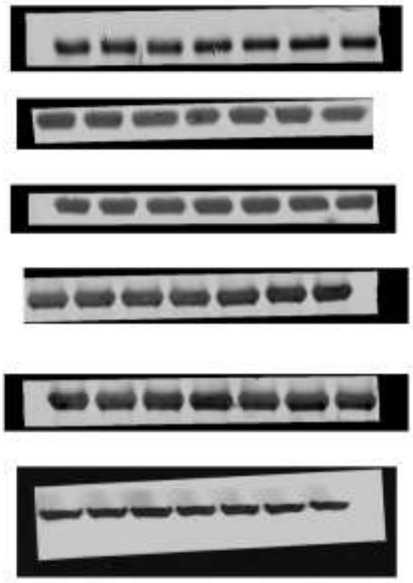

MFGE8

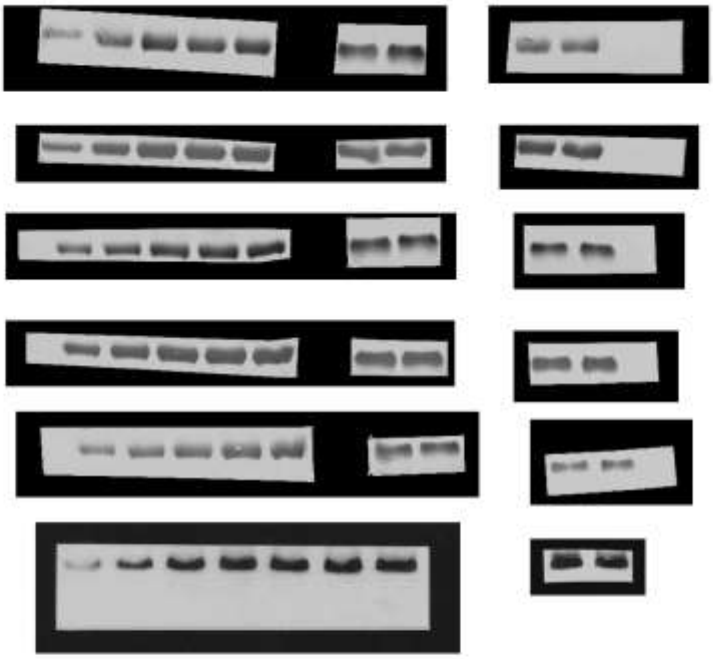

int-β3

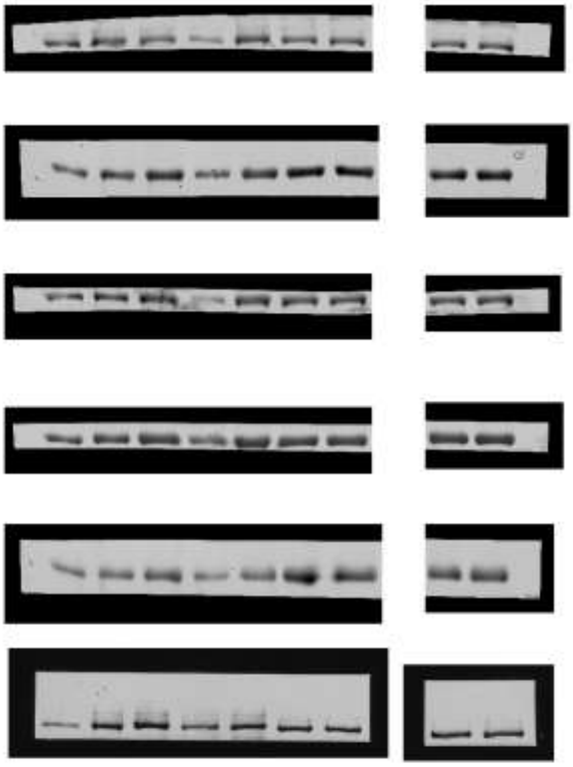

PI3K

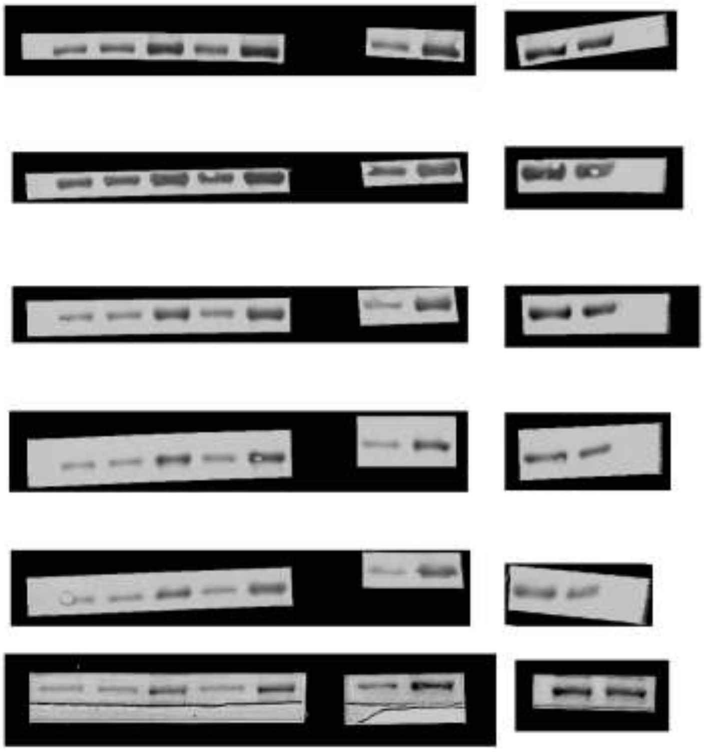

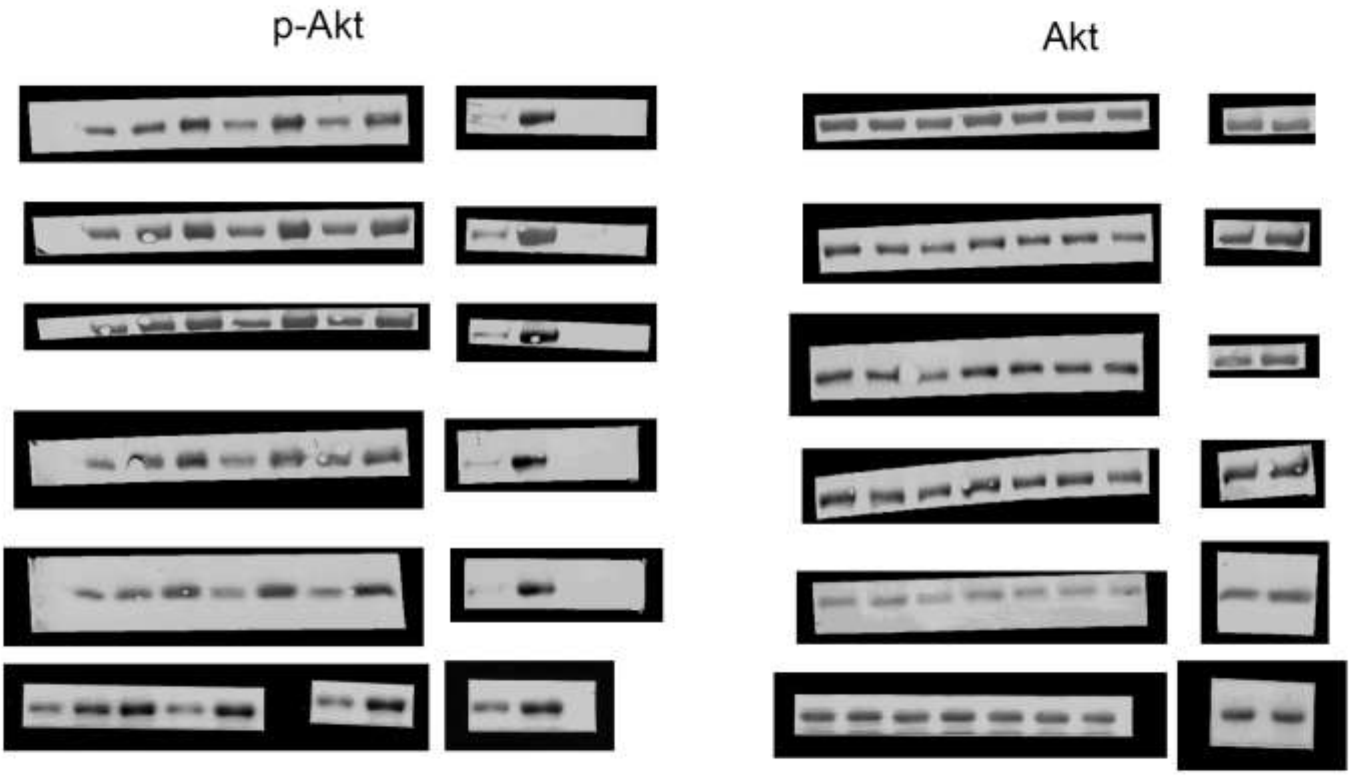
p-Akt
Akt

mTOR

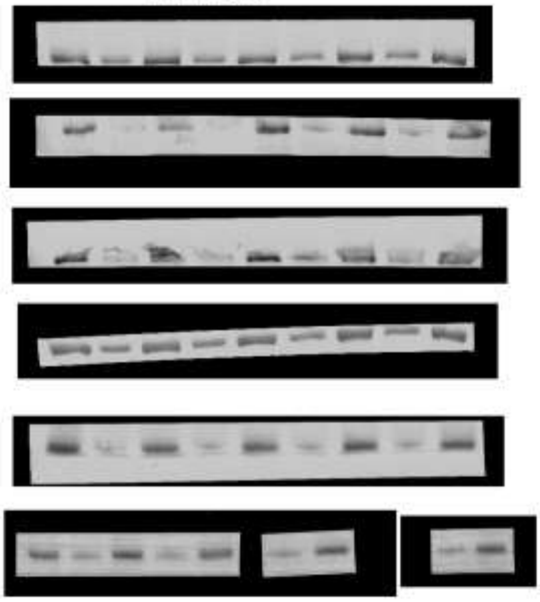

Cyclin D1

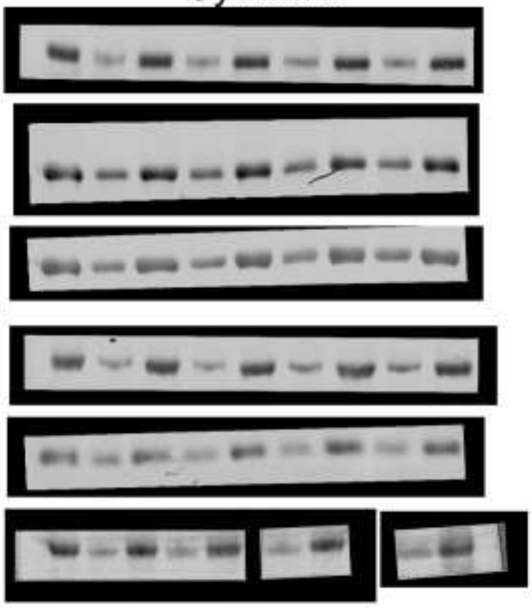

HPCA

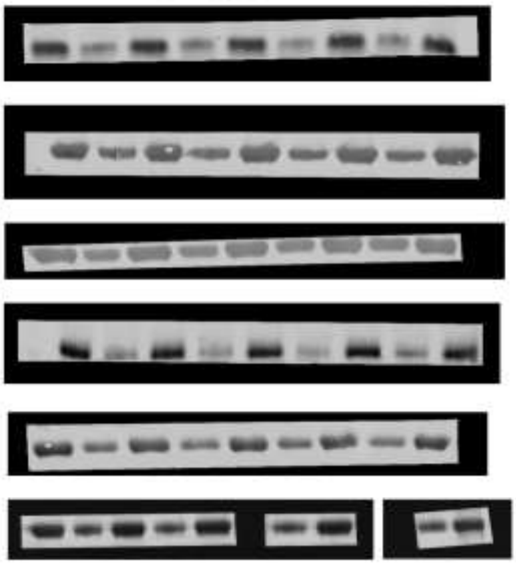

DCX

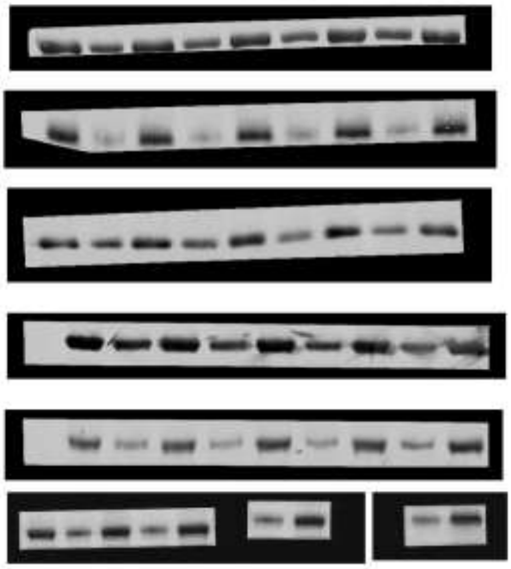

Actin

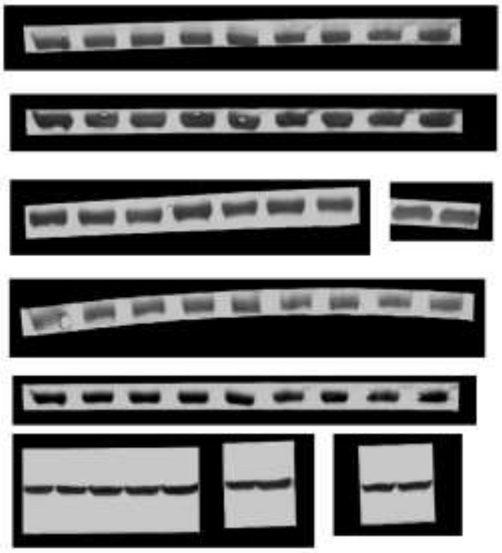

Supplement: Supplementary file 1 — Supplemental Materials [file 41420_2024_2132_MOESM1_ESM.pdf]
